# Assessment of the Mental Health of Police Officers: A Systematic Review of Specific Instruments

**DOI:** 10.3390/ijerph21101300

**Published:** 2024-09-28

**Authors:** Davi Oliveira Teles, Raquel Alves de Oliveira, Anna Luísa de Oliveira Parnaíba, Mariana Araújo Rios, Melissa Bezerra Machado, Priscila de Souza Aquino, Purdenciana Ribeiro de Menezes, Samila Gomes Ribeiro, Paula Renata Amorim Lessa Soares, Camila Biazus Dalcin, Ana Karina Bezerra Pinheiro

**Affiliations:** 1 Nursing Graduate Program, Federal University of Ceará, Fortaleza 60430-160, Brazil; enfdaviteles@alu.ufc.br (D.O.T.); queloliveiraa@alu.ufc.br (R.A.d.O.); priscilaaquinoenf@ufc.br (P.d.S.A.); purdenciana.menezes@sap.ce.gov.br (P.R.d.M.); samila@ufc.br (S.G.R.); renatalessa@ufc.br (P.R.A.L.S.); ana_karina@ufc.br (A.K.B.P.); 2 Programa de Educação Tutorial (PET), Nursing Department, Federal University of Ceará, Fortaleza 60430-160, Brazil; annaluisaparnaiba@alu.ufc.br (A.L.d.O.P.); marianarios@alu.ufc.br (M.A.R.); melissabezerramachado@alu.ufc.br (M.B.M.); 3 School of Health Sciences, University of Dundee, Dundee DD1 4HN, UK

**Keywords:** mental health, police, systematic review, surveys and questionnaires

## Abstract

Objective: The objective was to identify validated instruments from the literature that assess the mental health of police officers. Methods: This is a systematic review of validated instruments used to assess the mental health of police officers. Searches were conducted in the MEDLINE, Web of Science, Scopus, Embase, CINAHL/EBSCO, and Virtual Health Library databases. This review follows the JBI Manual for Systematic Reviews and the PRISMA statement. The methodological quality of the articles and the risk of bias were assessed. Results: A total of 1530 studies were identified across the six databases, with 158 studies read in full by the authors after excluding duplicates and those that did not meet the inclusion criteria. The final 29 studies were analyzed for methodological quality and risk of bias using the AXIS and SFS-D tools. Conclusion: This review identified 27 self-administered validated instruments useful for assessing various mental health outcomes in police officers, with the most frequently used being the Police Stress Questionnaire. These findings may help guide security force administration, occupational health professionals, and mental health researchers in selecting and implementing psychometrically reliable instruments for screening the mental health of police officers.

## 1. Introduction

Work is an intrinsic part of human life in society, influencing various aspects of social well-being, such as quality of life, health, dignity, and overall well-being. A harmonious relationship between the worker and their job is crucial, as the worker’s health impacts their performance, and the characteristics of the work environment affect the worker’s health [1].

In the context of essential workers’ mental health, these professionals face specific challenges that increase their risk of developing conditions detrimental to their well-being and mental health. While professionals like doctors, nurses, and firefighters also encounter significant occupational stressors, police workers have frequent encounters with violence and danger, added to similar challenges faced by other essential workers, such as heavy workloads and organizational challenges. These work challenges contribute to poorer mental health outcomes for police officers [2]. These factors can have distinct mental and emotional impacts, underscoring the need for studies focused on this population.

Research on police officers’ mental health indicates that these professionals experience elevated levels of operational stress, burnout, distress, and depression, which adversely affect both their personal lives and their job performance [3,4]. Therefore, addressing the mental health of police officers is critical, as this career presents high psychological risks, and mental health impairments can weaken their ability to manage public security responsibilities effectively [5].

Additionally, a meta-analysis estimated the global prevalence and risk factors for mental health problems among police officers, revealing a prevalence of 14.6% for depression, 14.2% for Post-Traumatic Stress Disorder (PTSD), 9.6% for generalized anxiety disorder, and 8.5% for suicidal ideation [6]. This situation can be attributed to the inherent characteristics of police work, including exposure to traumatic events and distressing circumstances, as well as a culture of bullying, lack of managerial support, and stigma surrounding mental health. These factors discourage officers from seeking help, leading to isolation and untreated conditions that either cause or exacerbate mental health problems [7,8,9].

Prior research on the mental health of police officers, including two systematic reviews, suggests that individual, organizational, and operational factors influence the prevalence of adverse psychological outcomes. These reviews emphasize that future research should focus on effective interventions and monitoring programs, as well as on comprehensively evaluating the psychological risks faced by police officers, considering individual, organizational, and operational factors [6,9].

To address these recommendations, the early detection and continuous monitoring of signs, symptoms, and behaviors that indicate the risks of developing or worsening mental disorders are essential strategies for police personnel. Once these risks are identified, appropriate measures can be taken to reduce the psychological impacts of work and provide timely treatment, thereby aiding in coping and recovery [10].

Research has consistently found that first responders, such as officers, are more likely than the general population to experience psychological distress as a result of their work. They are also less likely to seek professional help. This study also notes that common mental health screening programs may fail to detect psychological vulnerability in this group [11].

When discussing the mental health evaluation of police officers, it is important to note that research suggests that the psychological adaptation and vulnerability mechanisms in police officers differ from those in the general population [12]. Studies highlight that the mental health of police officers operates differently due to the unique combination of personality traits and psychological states influenced by the demands of their profession. These traits, along with the psychological states experienced on duty, develop over time in response to the occupational stressors of a police career [12].

In addition, a literature review pointed out that, despite the growing body of research studying occupational stress and mental health in police officers, researchers frequently use measurement instruments that have been developed based on other professional groups. These tools may not be well-suited to the unique demands of police work, such as emotional labor and physical risks. (Queirós et al., 2020) [3].

Thus, given the unique nature of police officers’ mental health traits, we highlight that mental health assessment tools specifically tailored to the police population are crucial, given the unique context in which police officers operate. Instruments designed for the general population may not adequately capture the distinct psychological challenges faced by police officers. Their highly stressful work environment, marked by exposure to violence, trauma, and life-threatening situations, significantly contributes to their elevated stress levels [13].

Despite these challenges, law enforcement culture often emphasizes stoicism, resilience, and the ability to manage stress independently. This fosters an environment where acknowledging mental health issues may be seen as a sign of weakness. The stigma surrounding mental health is further compounded by concerns about potential career repercussions, such as being deemed unfit for duty or losing advancement opportunities. As a result, police officers may be less likely to disclose the full extent of their psychological distress during clinical assessments, hindering accurate diagnosis and effective treatment [11]. The use of appropriate instruments could help mitigate this underreporting effect.

Given this context, the following research question arises: What are the validated instruments for assessing the mental health of police officers that have been psychometrically tested with a significant sample of officers? Considering the seriousness of mental health issues in this population and the lack of adequate assessment tools, a systematic review is essential to synthesize the available evidence and guide future research and interventions. By identifying these targeted instruments, we can make significant contributions to health policies aimed at police officers, enabling a better understanding of the factors that contribute to their mental health deterioration.

The relevance of this study lies in the fact that, to date, no systematic analysis has been conducted on instruments specifically designed to assess diverse mental health outcomes in police officers. Among the key reasons for conducting this study is the need to examine the psychometric properties of these assessment tools in different populations. Additionally, no prior research has identified validated instruments for assessing the mental health of police officers that have been psychometrically tested with a significant number of officers.

This study aims to identify the validated instruments that are used for assessing the mental health of police officers in the existing literature.

## 2. Methods

This systematic literature review was conducted in accordance with the Joanna Briggs Institute’s (JBI) guidelines. The following steps for systematic reviews of measurement instruments were followed: (1) formulation of the review question, (2) definition of inclusion and exclusion criteria, (3) development of the search strategy, (4) selection of studies, (5) assessment of methodological quality, (6) data extraction, and (7) data synthesis [14]. This review protocol is registered in the PROSPERO database for systematic reviews (registration number CRD42024506068).

Regarding the authors’ participation in the review process, D.O.T., R.A.O., A.L.O.P., M.A.R., and M.B.M. served as reviewers for the screening, quality assessment, and data extraction phases. Each independently provided their opinions on the inclusion/exclusion of studies and assessed the studies’ quality and data extraction. P.S.A., P.R.M., S.G.R., and P.R.A.L.S. contributed to the review conceptualization and search strategy design, and acted as supervisors for data extraction, resolving disagreements in study selection and quality assessment phases. P.S.A., C.B.D., and A.K.B.P. also served as review supervisors and participated in manuscript writing and reviewing, while being available to resolve any disputes during study selection and quality assessment.

### 2.1. Search Strategy

The steps of database searching, study selection, methodological quality assessment, data extraction, and synthesis were performed by five trained researchers working in pairs, who collectively analyzed the articles retrieved from the literature. The research question was developed using the Theme, Qualifier, Object (TQO) framework, with “mental health” as the theme, “assessment instruments” as the qualifier, and “police officers” as the object [15]. The following research question was posed: “What are the validated instruments for assessing the mental health of police officers that have been psychometrically tested with a significant number of officers?”.

A comprehensive research question and search strategy were used to ensure broad results, with study selection based on the inclusion and exclusion criteria outlined below. The search strategies employed natural language terms combined with controlled vocabulary from Health Sciences Descriptors (DeCS) and Medical Subject Headings (MeSH). Database searches were conducted in February 2024 across the following platforms: Medical Literature Analysis and Retrieval System Online/National Library of Medicine (MEDLINE), Web of Science, Scopus, Embase, Cumulative Index to Nursing and Allied Health Literature (CINAHL-EBSCO), and the Virtual Health Library (VHL), which includes databases such as the Latin American and Caribbean Health Sciences Literature (LILACS). The specific search strategies for each database are detailed in Table 1.

### 2.2. Inclusion and Exclusion Criteria

The inclusion criteria were as follows: (1) articles published in indexed journals, (2) studies utilizing validated and specific instruments to assess the self-reported mental health of the police population, and (3) studies validating general self-reported instruments applicable to this population. Study protocols and conference abstracts without complete data on the questionnaires were excluded. There were no language restrictions.

### 2.3. Screening and Quality Appraisal

Study selection was based on the criteria outlined above. The EndNote Web reference manager was employed for archiving, organizing, identifying, and removing duplicates. After removing duplicate entries, five reviewers individually analyzed the titles and abstracts using pre-established selection and screening procedures. At this stage, the final decision alignment among the five reviewers was 76%, which fell short of the expected 80% agreement, as recommended by the JBI reference [14]. Therefore, a joint meeting was arranged between the reviewers and experienced supervisors to discuss the discrepancies and review the inclusion and exclusion criteria.

After the aforementioned meeting, the authors agreed to include 158 studies for full-text reading. The five reviewers applied the same criteria for inclusion and exclusion and reached a 92% agreement at this stage. Any articles that did not receive a consensus among the reviewers (at least four out of five) were reviewed by a supervisor. The supervisor would then decide whether to include or exclude them for the next stage.

**Figure 1:**
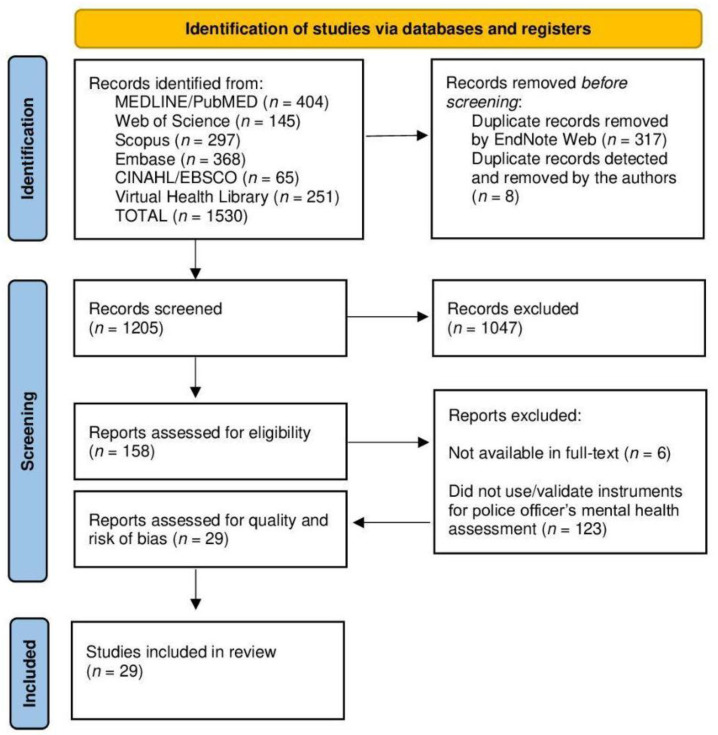
Flowchart of the study selection process. Source: Adapted from PRISMA checklist by the authors, 2024.

Following the JBI manual and PRISMA guidelines, a flowchart detailing the study selection process was created, as shown in Figure 1.

The Appraisal Tool for Cross-Sectional Studies (AXIS tool) was employed to evaluate the methodological quality and risk of bias in the observational studies. This tool, consisting of 20 items, is designed to critically assess the quality, study design, and potential bias, with responses categorized as “yes”, “no”, or “do not know” [15]. Although the AXIS tool does not provide a numerical scale for stratifying study quality, requiring some subjectivity in evaluation, using numerical ratings can be problematic as the outputs are not uniform and difficult to interpret, limiting their predictability for assessing study quality [16]. Following another JBI-based systematic review, the five reviewers completed the 20 AXIS questions that were each scored by assigning numeric values: “Yes” (scored as 1) and “No” or “Do not know” (scored as 0), resulting in a general score from 0 to 100% of compliance [17].

For the assessment of methodological studies, the SFS scoring system version D (SFS-D) was applied. This instrument evaluates the theoretical foundation and rigor of surveys and questionnaires using 29 items covering the instrument’s purpose, methodological rigor, and general considerations. The quality of the articles was classified as weak (0–25%), moderate (26–50%), strong (51–75%), or excellent (76–100%) [18].

After all scores were completed, a meeting was held to help establish consensus (at least 80% agreement between reviewers) in the quality appraisal scores; a supervisor resolved any disagreements. Finally, the reviewers included those that met at least 51% of the SFS-D items—which stands for at least strong quality studies [19]—and at least 70% in the AXIS tool’s calculated score, which would stand for high-quality studies according to previous research [17]. It is important to note that there was a high level of consensus at this stage (>80%), and no study was excluded.

### 2.4. Data Extraction and Synthesis

The data extraction process followed the guidelines of the Preferred Reporting Items for Systematic Reviews and Meta-Analyses (PRISMA) and the JBI manual for reviews of measurement instruments [14,20]. Information on study characteristics (authors, year of publication, methodology, sample, and country) and instrument details (analyzed construct, presence and number of subscales, number of items, cutoff scores, validation statistics such as Cronbach’s alpha/internal consistency, and availability of translations) was extracted individually by the authors and recorded in a Google Sheets spreadsheet.

The results were synthesized and discussed narratively and through illustrative tables and charts to enhance clarity and readability. In this article, the reviewed studies are referenced using codes corresponding to the letter “S” for study, followed by the number assigned to each study in Table 2. Ethical review and approval were not required for this study, as it is a systematic review and meta-analysis based on publicly accessible data from databases.

## 3. Results

The search strategies resulted in 1530 studies from six databases. After removing duplicates and excluding articles that did not meet the inclusion criteria, the authors thoroughly reviewed 158 studies.

### 3.1. Quality Assessment of the Included Studies

Twenty-nine studies were evaluated for methodological quality and risk of bias using the AXIS and SFS-D tools, and all of them were included in the final sample. Among the observational studies assessed with the AXIS tool, four articles scored 90%, six scored 85%, and eight scored 80%, all above the cutoff. For the SFS-D scoring system, which focuses on instrument validation or development, four studies were classified as “strong” quality, meeting at least 51.6% of the SFS-D criteria, while nine studies were classified as “excellent” quality, with scores ranging from 75.8% to 87%. The average score was 76.17%.

### 3.2. Characteristics of the Included Studies

Detailed information on the 29 studies included in the review can be found in Table 2.

Twenty studies (69%) were published in the last five years (S4; S5; S7; S9; S11–S14; S18–S29), four in the last ten years (S1; S6; S8; S15), and the remaining studies were published within the last twenty-one years, with the oldest dating back to 2003 (S16). Fifteen studies employed a cross-sectional design (S1; S2; S4; S7; S9; S12; S13; S18–S20; S22–S24; S26; S29), while eleven were methodological studies. Of these, seven focused on validation (S5; S6; S8; S11; S15; S21; S25) and four focused on instrument development (S3; S10; S16; S17). Two studies utilized a mixed-methods design, combining cross-sectional and validation approaches (S14; S28), and one study used a cohort design (S27). All studies were available in English, except for S6 (Portuguese) and S9 (Croatian).

Regarding the sampling strategies for officers, 13 studies used convenience sampling. Within these, six employed web-based convenience sampling (S4; S10; S18; S19; S26; S29), four utilized multi-stage sampling (S9; S16; S23; S28), two used quota sampling (S11; S15), and one study each applied consecutive (S27) and universal sampling (S22). The sampling procedure was unclear in two studies (S3; S13).

Sample sizes ranged from 22 to 8088, with a mean of 996 subjects and a median of 269. The percentage of female officers was reported in 23 studies, ranging from 0% to 48.1%, with a mean of 22.1% and a median of 16.2%. The mean age of participants was reported in 22 studies, ranging from 29.27 to 42 years, with an overall mean of 37.3 years and a median of 38 years. Fifteen studies provided data on years of service, ranging from 7 to 19 years, with a mean of 11.1 years and a median of 10 years.

Among the included studies, three involved other public safety personnel (S4; S21; S23), such as paramedics, fire and rescue professionals, and state emergency services personnel, while the remaining studies focused exclusively on police officers. Of the 23 studies that reported on officer types, 10 included multiple categories (e.g., coast guard, federal police, correctional officers), whereas the others focused on a single type of officer (Table 2).

Six studies were conducted across 18 countries: in Asia (S12, S22 in India; S13 in Malaysia; S24 in South Korea; S25, S29 in China), North America (S16, S17 in the USA; S4; S19; S20; S21 in Canada), Latin America and the Caribbean (S28 in Brazil; S7 in Mexico; S9 in Trinidad and Tobago), and the remainder in Europe.

### 3.3. Characterization of the Instruments Identified in the Review

From the 29 reviewed articles, 27 instruments for assessing police officers’ mental health were identified. These instruments were categorized based on the mental health constructs they assessed: work-related stress (*n* = 8), Burnout (*n* = 4), coping and job satisfaction (*n* = 6), risk of psychological distress (*n* = 3), and mental health disorders (*n* = 6). The number of items in the instruments ranged from 1 (BDI-II) to 90 (Symptom Checklist 90), with a mean of 24, a standard deviation of 20, and a median of 20 (interquartile range: 12). Additional details are provided in Table 3.

### 3.4. Psychometric Properties of the Instruments Identified in the Review

In addition to structural properties (see Table 3), this review also collected data on the psychometric properties of the 27 questionnaires reported in the studies (see Table 4). Cronbach’s alpha was the most frequently reported coefficient, being included in all studies. Test–retest reliability was reported in nine studies, and inter-item correlation appeared in six studies. Other coefficients were less common: the split-half coefficient was reported for two questionnaires, PMHA (0.85) and TIES-r (0.71); Spearman–Brown for VBBI (0.859); and McDonald’s Omega for DECORE-21 (0.8). The lowest Cronbach’s alpha was 0.6 for DECORE-21′s cognitive demands subscale, while the highest were 0.94 for VBBI, PSQ-Op, and PSQ-Org.

### 3.5. Work-Related Stress Instruments

Eight instruments (29.6%) assessed work-related stress: the Norwegian Police Stress Survey (NPSS), the Job Stress Survey (JSS), the Effort-Reward Imbalance Questionnaire (ERIQ), the Law Enforcement Officer Stress Survey (LEOSS), the Operational Police Stress Questionnaire (PSQ-Op), the Organizational Police Stress Questionnaire (PSQ-Org), the Perceived Stress Scale (PSS), and the Police Mental Health Abilities (PMHA).

Among these, three instruments (JSS, ERIQ, PSS) were originally designed for the general population and were later validated for police officers. Item counts ranged from 10 (PSS) to 36 (NPSS), and all questionnaires utilized Likert-type scales. The lowest Cronbach’s alpha was 0.78 (ERIQ), while the highest was 0.94 (PSQ-Op and PSQ-Org). Only two instruments (JSS, ERIQ) had described cutoff scores. The most frequently cited instruments were PSQ-Op (eight studies) and PSQ-Org (six studies).

### 3.6. Burnout Instruments

Four instruments (14.8%) assessed burnout: the Maslach Burnout Inventory (MBI), the Granada Burnout Questionnaire (GBQ), the Spanish Burnout Inventory (SBI), and the V. Boyko Burnout Inventory (VBBI). All used Likert-type scales, with item counts ranging from 20 (SBI) to 84 (VBBI). Cronbach’s alpha values ranged from 0.60 to 0.94. Only two instruments (SBI, VBBI) provided cutoff scores. All instruments included subscales ranging from 3 to 4 dimensions. The most frequently cited were the MBI (six citations) and SBI (three citations).

### 3.7. Coping and Satisfaction with Work Instruments

Six questionnaires (22.2%) evaluated workplace satisfaction and coping/resilience strategies related to police work: the Work and Well-Being Assessment for Police (WWBAP), the Police Role Expectations (PRE), the Brief COPE, the Abridged Job Descriptive Index (JDI), the Brief Resilience Scale (BRS), and the Copenhagen Psychosocial Questionnaire (COPSOQ).

All instruments used ordinal scales, with the shortest having six items (Brief COPE) and the longest containing 46 items (WWBAP). Cronbach’s alpha values were high for all instruments. Some assessed police officers’ expectations regarding their job and its impact on their mental health, while others focused on general job satisfaction, physical and psychological health, and interpersonal relationships. Two instruments (TBC and BRS) specifically evaluated resilience, which can influence job satisfaction. The most cited instruments were the Brief COPE and BRS (three studies each).

### 3.8. Risk of Psychological Distress Instruments

Two instruments (7.4%) assessed the risk of psychological distress: the Psychological Injury Risk Indicator (PIRI) and the DECORE-21. The PIRI inquired about the impact of work on routine aspects such as sleep, rest, energy, tiredness, self-medication, and stress. The DECORE-21 evaluated psychological risk through questions about cognitive demands, work support, and personal control. Additionally, one instrument (3.7%) assessed the risk of suicidal ideation using a single question from the Beck Depression Inventory (BDI-II). None of these instruments were specifically designed for police officers.

All instruments employed Likert-type scales. Validation statistics indicated that all instruments had Cronbach’s alpha values greater than 0.60. The number of items ranged from 1 (BDI-II) to 30 (PIRI). Among these, two instruments included subscales within their constructs. The DECORE-21 was the most cited instrument in this category, appearing in two studies.

### 3.9. Mental Health Disorders Instruments

Six instruments (22.2%) assessed minor psychiatric disorders: the Hospital Anxiety and Depression Scale (HADS), the Patient Health Questionnaire (PHQ-8), the Generalized Anxiety Disorder Scale (GAD-7), the Self-Report Questionnaire (SRQ20), the Symptom Checklist 90 (SC-90), and the Impact of Event Scale—Revised (IES). The number of items ranged from 7 (GAD-7) to 90 (Symptom Checklist 90). All instruments achieved Cronbach’s alpha values greater than 0.70, with the highest alpha being 0.93 for the Symptom Checklist 90. All instruments reported cutoff scores, and three of them included subscales. The most cited instruments in this category were the PHQ-9 (four studies) and the GAD-7 (three studies).

## 4. Discussion

The present study aimed to identify the existing validated instruments for assessing the mental health of police officers, a population particularly vulnerable to unique occupational stressors such as exposure to confrontation, violence, and traumatic incidents, including the risk of injury or death [50]. While many studies focus on the mental health of public safety personnel (PSP) and police officers and their associated factors, there is a notable lack of analysis regarding the questionnaires available for evaluating and detecting these mental health outcomes. This systematic review addresses this gap by providing a comprehensive overview of validated and appropriate instruments specifically designed for assessing mental health symptoms among police officers.

Despite the range of existing mental health assessment instruments, it is crucial to evaluate their content validity and suitability for use in health contexts, particularly given the specific demands of police work. Many of the identified instruments were developed specifically for police officers, while others were general instruments validated or used within this population. This distinction is important because validating existing instruments for a particular population allows for the observation of occupational group differences and the investigation of specific stressors associated with each profession. Using instruments tailored to a given population is essential, as a previously validated instrument may not perform well if applied to a different group from the one for which it was originally developed [51].

Although validating general public questionnaires for police officers is a viable approach, specific instruments designed for this population enable a more accurate assessment of their unique needs and vulnerabilities. Police officers are consistently exposed to stressors inherent to their roles, such as frequent contact with violence, irregular sleep patterns, lack of support from superiors, and emotional exhaustion. These factors contribute to a higher risk of developing work-related problems that generic questionnaires may not adequately assess [50].

Analyzing the included studies, we found that the majority focused exclusively on police officers, encompassing various duties such as conservation, judicial, traffic, correctional, frontline, and detective roles from both rural and urban areas. This analysis is crucial due to the unique stressors associated with different police workplaces, which contribute to varying levels of exposure to violence, shift schedules, physical activity, confrontation, and work-related stress [52]. These factors must be considered when selecting a questionnaire, as some instruments may not adequately capture the psychological demands faced by different subgroups within the police force.

In our review, ten studies used or validated instruments across mixed types of police work categories, but none adapted or accounted for specific differences among various types of police officers. This oversight may be problematic, as the different roles within police work involve varying levels of exposure to stressors and occupational risks. Tailoring assessment instruments to the specific tasks and roles of different police officers could enhance diagnostic accuracy and lead to more effective interventions.

Additionally, few studies included other PSP alongside police officers for mental health assessment. For example, study S4 found significant differences in organizational and occupational stress between police officers and other professions, with police officers experiencing the highest levels of stress [24]. Similarly, study S23 identified substantial differences between police officers and other PSP, noting that police officers had the lowest well-being, highest psychological distress, highest rate of mental health conditions, lowest resilience, and the highest binge drinking rate [43].

Thus, while police officers face unique stressors compared to other PSP and among different police roles, these differences were not highlighted in the included studies. Recognizing and addressing these disparities is essential for developing tailored and effective mental health assessments and interventions.

Regarding the reliability and validity of the analyzed instruments, it was observed that most studies assess internal consistency using Cronbach’s alpha coefficient, while fewer address other forms of validity, such as external, criterion, and convergent validity. Although internal consistency remains a crucial aspect of reliability [53], other types of validity are equally important to ensure that instruments not only measure constructs consistently, but also predict relevant outcomes and correlate adequately with related measures [54]. Therefore, we recommend that researchers continue developing and validating these instruments across various settings using various psychometric analyses, including criterion, and convergent validity. It is also important for researchers to report these aspects clearly and transparently to enhance the confidence and understanding of their findings.

Criterion validity is crucial, as it shows the relationship between the quality of the instrument and an appropriate external “gold standard”. It takes into account sample size, sensitivity, specificity, and predictive values. This validation ensures that the instrument effectively and accurately measures what it intends to measure [55,56]. On the other hand, convergent validity assesses whether the questionnaire aligns with other established measures of the same construct, ensuring it consistently reflects the mental health issues it aims to evaluate [57].

Furthermore, other studies highlight other types of content validation with robust methods, such as the Content Validation Template (CVT), which is used considering raw data input and the execution of a simple operation with dedicated computation for content validation [58]. These validity measures collectively help establish the questionnaire’s reliability and suitability for clinical or research settings.

All identified instruments were self-administered. This approach is partly due to the stigma associated with mental illness, which is particularly pronounced in the police sector. Police professionals often feel pressured to maintain resilient postures and minimize displays of vulnerability [59]. This cultural stigma can significantly influence both the choice of mental health assessment questionnaires and the interpretation of the data obtained. Firstly, this stigma may lead to the selection of instruments that prioritize the assessment of more “acceptable” or less stigmatized symptoms, such as stress or fatigue, rather than issues more directly related to mental disorders, like depression or anxiety [60]. The preference might also lean towards shorter and less-intrusive questionnaires, aiming to reduce the respondents’ discomfort in disclosing deeper emotional concerns [60].

Additionally, the stigma surrounding mental health can influence how the data are understood. Police officers, who may be hesitant to disclose their emotional challenges due to concerns about the repercussions, might provide inaccurate or minimized responses. This could result in underestimating the actual prevalence of mental health symptoms [11]. Consequently, the findings may inaccurately portray higher levels of well-being, leading to biased conclusions about the group’s mental health [11]. Therefore, it is essential for researchers to consider the impact of this stigma when analyzing the results. It may also be beneficial to use qualitative methods in addition to quantitative data to gain a better understanding of the responses and identify potential response biases.

From another perspective, despite the resistance of police officers to disclose symptoms due to stigma, self-reporting questionnaires have their own limitations. While some of the literature supports the constructed and ecological validity of self-administered neuropsychological tests compared to traditional testing [61], others highlight that the lack of supervision during administration can compromise data accuracy, as participants might minimize or omit symptoms and lack immediate feedback for questions [62]. Furthermore, stigma can affect not only the use of the instruments, but also the interpretation of results. Implementing strategies to mitigate these effects, such as creating more supportive assessment environments and reducing stigma around mental health among police officers, is essential to ensure the effectiveness of interventions.

The number of items in the reviewed questionnaires ranged from 20 to 84, which is a critical factor in selecting the most effective instrument. Well-designed questionnaires are essential for obtaining relevant information efficiently, and should generally contain between 25 and 30 items and be completed within 30 min to maintain participant engagement. If a questionnaire is too lengthy, it is advisable to divide it into sections and provide alternatives to mitigate the risk of incomplete responses. From this perspective, as the number of questions in a questionnaire increases, there is a tendency for participants to speed up or resort to satisficing, which severely impacts the quality, reliability, and response rates. In the case of a long questionnaire, i.e., more than 30 questions, a higher amount of missing data or non-responses must be anticipated, and provisions should be made to address these issues [63].

Previous studies have shown that participants were more likely to report being careless when responding to longer questionnaires (60 items per measurement occasion) compared to shorter questionnaires (30 items per measurement occasion) [64]. Studies also suggest that longer questionnaires require more cognitive effort from participants, leading them to potentially use shortcuts or response styles to reduce the cognitive load [65].

These recommendations and experiences align with our findings, as many of the included studies reported low response rates among police officers.

Consistent with other systematic reviews on police officers’ mental health, our review identified a range of outcomes, including burnout, stress, psychological injury, suicidal ideation, anxiety, depression, PTSD, and others [6,9]. Adverse psychological predictors identified in our study include the following: the imbalance between psychological and emotional demands and the lack of positive, rewarding interactions; exposure to violence; high demands coupled with low control; irregular shift schedules; and toxic organizational cultures. Furthermore, the risk of harm or injury and witnessing others’ suffering can accumulate stress over time. Officers who perceive their work as lacking positive outcomes or rewards may become disengaged, performing only the minimum required duties and avoiding additional effort or personal initiative.

Additionally, it is important to consider the impact of cultural and regional factors on the application and validity of the instruments. Our review highlighted the diverse geographical contexts in which the studies were conducted, emphasizing the need to adapt instruments to specific cultural and regional realities through cross-cultural methods. Policing practices vary significantly across countries due to differences in location, population needs, organizational structures, infrastructure, available resources, and technology [66]. Furthermore, variations in police officer categories, workloads, duties, wages, social prestige, and sociopolitical challenges can directly affect the effectiveness of assessment instruments [66].

It is also important to highlight that even within the same country, differences in police work between rural and urban areas can affect operational and cultural aspects of policing, as well as the unique risks faced by rural officers [67]. Therefore, future studies should prioritize the cross-cultural adaptation of research instruments to ensure their validity and reliability across diverse cultural and regional contexts. This process involves modifying instruments to retain their original meaning and measurement properties while making them culturally relevant and understandable to the target population [68].

In terms of practical implications, the findings of this review can guide healthcare professionals, policymakers, and security teams in developing more-effective assessment strategies. Police officers encounter unique stressors that significantly impact their mental health, making the careful selection of appropriate instruments crucial for the early detection of mental distress and implementation of preventive interventions. Additionally, the development of public policies to support police officers’ mental health could benefit from these findings, reinforcing the need for evidence-based practices to promote the well-being of this workforce.

This review is also valuable for researchers in mental and occupational health, particularly within police and public safety contexts. It identifies a range of instruments for future research and provides insights into various outcomes, cutoffs, and psychometric properties. These instruments can be utilized in various research designs and may stimulate cross-cultural adaptations, validation, and applications in different countries and settings.

This study has some limitations. First, the review was restricted to studies available in the selected databases and peer-reviewed publications, potentially excluding relevant data from gray literature. According to AMSTAR—Assessing the Methodological Quality of Systematic Reviews—an important criterion for a systematic review is the search for sources of gray literature when relevant to the study, aiming to cover all of the knowledge on a given topic [69]. However, the study did not include sources of gray literature. Because of that, this review may miss important perspectives or underreport relevant findings, particularly in emerging or under-researched areas, potentially affecting the generalizability of our conclusions. Also, the unavailability of some full-text articles may have further limited the inclusion of potentially relevant studies. Future research should expand the scope to include gray literature and additional sources to achieve a more comprehensive understanding of the available instruments.

In conclusion, this study contributes significantly to the field by synthesizing the evidence on validated instruments for assessing police officers’ mental health. Nonetheless, the gap in studies focusing on different contexts and the variability among police forces underscore the need for further research. Given the unique characteristics of police work and the diverse roles of police officers, it is important to have instruments adapted to each context. There is also the stigma that may cause underreporting of symptoms [11]. To address this, it is recommended to conduct longitudinal studies that include additional contextual variables and cross-validate instruments across different police populations. This will help in developing reliable and customized psychological assessment tools for this population.

## 5. Conclusions

The review identified 27 validated self-administered instruments for assessing police officers’ mental health. These instruments featured subscales addressing various mental health components, including work-related stress, burnout, coping, job satisfaction, risk of psychological injury, and mental health disorders. This underscores the fact that police officers’ mental health is shaped by a complex interplay of individual, organizational, and operational risks.

In summary, the Police Stress Questionnaire—Operational and Organizational emerged as the most frequently used instrument for measuring occupational stress in the studies reviewed. It demonstrated the highest Cronbach’s alpha and strong item–item correlations. The Maslach Burnout Inventory was the most utilized for burnout assessment, with the V. Boyko Burnout Inventory showing the highest internal consistency. In the domain of coping, the Brief COPE and Brief Resilience Scale were notable for their brevity and strong psychometric properties, including good Cronbach’s alpha and test–retest reliability. Regarding mental health disorders, the Patient Health Questionnaire—9 and Generalized Anxiety Disorder—7 were the most cited, with both demonstrating excellent Cronbach’s alpha and test–retest reliability in police populations.

The findings from this review provide valuable insights for mental health professionals, occupational clinicians, policymakers, police administrators, security forces’ health staff, and researchers. They can guide the implementation of effective screening and monitoring strategies using high-quality psychometric instruments, ultimately contributing to the development of targeted interventions. Future research should emphasize cross-cultural validation and consider the diverse contexts of police forces to enhance the applicability and robustness of psychometric tools for this population.

## Figures and Tables

**Table 1:** Search strategies adapted for each database.

Database	Search Strategies
MEDLINE/PubMed	((Police) AND (Mental health)) AND (Surveys and Questionnaires)
Web of Science	((ALL = (Police)) AND ALL = (Mental health)) AND ALL = (Surveys and Questionnaires)
Scopus	((police) AND (mental AND health) AND (surveys AND questionnaires))
Embase	‘police’ AND ‘mental health’ AND ‘surveys and questionnaires’
CINAHL (EBSCO)	police AND mental health AND (surveys and questionnaires)
Virtual Health Library (VHL)	“Police” AND “Mental health” AND “Surveys and Questionnaires”

Source: Prepared by the authors, 2024.

**Table 2:** Characteristics of the included studies (*n* = 29).

Code/Reference	Origin	Study Design	Sample Description	Sample Size (*n*)	Instruments
S1 Maran et al. 2015 [21]	Italy	Cross-sectional	Unit managers, non-commissioned officers, emergency officers, and traffic patrol officers	617	Brief COPE; Police Stress Questionnaire-Operational and Organizational (PSQ-OP and PSQ-Org)
S2 Berg et al. 2006 [22]	Norway	Cross-sectional	Investigation police, uniformed officers, and administration officers	3272	Norwegian Police Stress Survey (NPSS); Job Stress Survey (JSS); Maslach Burnout Inventory (MBI)
S3 Berg et al. 2005 [23]	Norway	Methodological instrument creation	Police officers	3272	Job Stress Survey (JSS) and Norwegian Police Stress Survey (NPSS)
S4 Carleton et al. 2020 [24]	Canada	Cross-sectional	Correctional officers, federal police, municipal/provincial police, public safety communications officials	3118	PSQ-Op; PSQ-Org and Perceived Stress Scale (PSS)
S5 Fuente-Solana et al. 2020 [25]	Spain	Methodological instrument validation	Officers, middle managers, and managers	1884	Maslach Burnout Inventory (MBI); Granada Burnout Questionnaire (GBQ)
S6 Figueiredo-Ferraz et al. 2014 [26]	Portugal	Methodological instrument validation	Public safety police	245	Spanish Burnout Inventory (SBI)
S7 García-Rivera et al. 2020 [27]	Mexico	Cross-sectional	Preventive police officers	276	Spanish Burnout Inventory (SBI) and PSQ-Op
S8 Harizanova et al. 2016 [28]	Bulgaria	Methodological instrument validation	Correctional Officers	50	V. Bokyo Burnout Inventory (VBBI)
S9 Jelaš et al. 2020 [29]	Trinidad and Tobago	Cross-sectional	Police officers	331	MBI; PSQ-Op; PSQ-Org
S10 Juniper et al. 2010 [30]	United Kingdom	Methodological instrument creation	Officers, police community support officers, and civilian staff	822	Work and well-being assessment for police (WWBAP)
S11 Luceño-Moreno et al. 2021 [31]	Spain	Methodological instrument validation	Patrol officers, corporals, non-commissioned officers, police commissioner officers	217	Effort-Reward Imbalance Questionnaire (ERIQ); DECORE-21; MBI
S12 Maurya, 2019 [32]	India	Mixed methods	Civil police	203	Police role expectations (PRE)
S13 Mohamed et al. 2022 [33]	Malaysia	Cross-sectional	Police officers	1641	PSQ-Op and PSQ-Org
S14 Queirós et al. 2020 [34]	Portugal	Cross-sectional	National police	1131	PSQ-OP; PSQ-Org; Brief COPE and SBI
S15 Talavera-Velasco et al. 2018 [35]	Spain	Methodological instrument validation	Local police officers	223	DECORE-21
S16 Van Hasselt et al. 2003 [36]	United States of America (USA)	Methodological instrument creation	Detectives, traffic officers, SWAT	166	Law Enforcement Officer Stress Survey (LEOSS)
S17 Winwood et al. 2009 [37]	United States of America (USA)	Methodological instrument creation	Frontline police officers	217	Psychological Injury Risk Indicator (PIRI)
S18 Anders et al. 2022 [38]	Switzerland	Cross-sectional	Emergency police, judicial police, community police, administrative, traffic, special forces, and dispatch center	1073	The Impact of Event Scale—Revised (TIES-r); MBI; Brief COPE, The Hospital Anxiety and Depression Scale (HADS) and suicide ideation from the Beck Depression Inventory—II (SI-BDI-II).
S19 Andrews et al. 2022 [39]	Canada	Cross-sectional	Coast Guard and Conservation Officers	412	General Anxiety Disorder-7 (GAD-7) and Patient Health Questionnaire-9 (PHQ-9)
S20 Beshai et al. 2022 [40]	Canada	Cross-sectional	Royal Canadian Mounted Police	173	Job Descriptive Index (JDI); PHQ-9; GAD-7; Brief Resilience Scale (BRS); Perceived Stress Scale (PSS)
S21 Huang et al. 2022 [41]	Canada	Methodological instrument validation	Police officers	22	MBI; PHQ-9; GAD-7; BRS; PSS
S22 Jeganish et al. 2024 [42]	India	Cross-sectional	State police	142	PHQ-9 and PSS
S23 Kyron et al. 2022 [43]	Australia	Cross-sectional	Police officers	8088	BRS
S24 Lee; Hans, 2022 [44]	South Korea	Cross-sectional	Police officers	269	TIES-r
S25 Liao et al. 2022 [45]	China	Methodological instrument validation	Police officers	767	Police Mental Health Ability (PMHA)
S26 Ohlendorf et al. 2023 [46]	Germany	Cross-sectional	Federal police	200	Copenhagen Psychosocial Questionnaire (COPSOQ) and PSQ-Op
S27 Rohwer et al. 2022 [47]	Germany	Cohort	Police officers	116	Copenhagen Psychosocial Questionnaire (COPSOQ)
S28 Tavares et al. 2022 [48]	Brazil	Mixed methods	Civil Police	237	Self-Reported Questionnaire-20 (SRQ-20)
S29 Wu et al. 2023 [49]	China	Cross-sectional	Security police, criminal police, constable police, community police, and internal work police	358	Symptom Checklist 90 (SCL-90)

Source: Elaborated by the authors, 2024.

**Table 3:** Characteristics of the identified instruments (*n* = 27).

Instrument	Outcomes	Subscales/Domains	Items	Cutoff Values	Used in	Police Specific?
Maslach Burnout Inventory (MBI)	Burnout.	Emotional exhaustion, depersonalization, and personal achievement.	22	None.	S2, S5, S9, S11, S18, and S21	No
Granada Burnout Questionnaire (GBQ)	Burnout.	Emotional exhaustion, depersonalization, and personal achievement.	26	None.	S5	No
Spanish Burnout Inventory (SBI)	Burnout.	Enthusiasm for work, psychic wear, indolence, and guilt.	20	Low scores on enthusiasm for work and high scores on psychic exhaustion and indolence represent high levels of burnout syndrome.	S6, S7, and S14	No
V. Bokyo Burnout Inventory (VBBI)	Burnout.	Stress, resistibility, and exhaustion.	84	Scoring is carried out in two stages. The first evaluates each symptom: <9 (symptom is not important), 10–15 (symptom settling in), and >16 (active symptom). After the sum of the symptoms, there are scores for each of the three phases, where <36 = the phase is not developed, 37–60 = in the process of development, and >61 = the phase is developed.	S8	No
The Job Stress Survey (JSS)	Occupational stress.	Pressure at work, lack of support.	30	Each stressor is evaluated on a Likert scale from 0 to 9+ points by frequency of occurrence in the last six months. The sum is the result of the scale.	S2 and S3	No
The Norwegian Police Stress Survey (NPSS)	Occupational stress.	Job pressure, lack of support, serious operational tasks, and work injuries.	36	None.	S2 and S3	Yes
Effort-Reward Imbalance Questionnaire (ERIQ)	Occupational stress.	Effort, reward (esteem, financial, status, and job security), and overcommitment.	23	Effort score from 6 to 30; reward from 11 to 55; overcommitment score from 6 to 30. The effort and overcommitment scores are added together, which indicate imbalance and occupational stress if they are higher than the reward score	S11	No
Law Enforcement Officer Stress Survey (LEOSS)	Occupational stress.	None.	25	None.	S16	Yes
Operational Police Stress Questionnaire (PSQ-OP)	Occupational stress.	None.	20	The higher the score, the greater the stress.	S1, S4, S7, S9, S13, S14, S18, and S26	Yes
Organizational Police Stress Questionnaire (PSQ-Org)	Occupational stress.	None.	20	The higher the score, the greater the stress.	S1, S4, S9, S13, S14, and S18	Yes
Work and Well-Being Assessment for Police (WWBAP)	Work-related well-being.	Advancement, home work interface, job, organizational, physical, psychological, relationships, workload, and facilities.	46	The higher the score, the lower the well-being.	S10	Yes
Police Role Expectations (PRE)	Expectations regarding the job.	Aggressiveness, facilitative, conformist, and authoritative.	18	The score from 18 to 90, the higher the score, the higher the job expectation.	S12	Yes
Psychological Injury Risk Indicator (PIRI)	Risk of psychological injury.	Turbulent sleep/poor sleep hygiene, maladaptive experience, chronic fatigue, consistent failure to recover physical and emotional energy, PTSD symptomatology, and alcohol abuse/self-medication related to stress.	30	A standardized PIRI score of more than 25 corresponded to possible psychological injury, while higher scores indicate a greater risk of injury.	S17	Yes
DECORE-21	Risk of psychological injury.	Cognitive demands, control, work organization support, and rewards.	21	The higher the score, the greater the psychological risk suffered at work.	S11 and S15	No
Brief COPE	Coping strategies.	Acceptance, active coping, guilt, behavioral withdrawal, denial, distraction, emotional expression, emotional support, humor, instrumental support, planning, positive reinterpretation, religion, and substance use.	28	On a four-point Likert scale, ranging from zero points to three points depending on the answer; higher scores reflect a higher tendency to implement corresponding coping strategies.	S1, S14, and S18	No
The Hospital Anxiety and Depression Scale (HADS)	Anxiety and depression.	Anxiety and depression.	14	The typical clinical threshold for the presence of anxiety is a score ≥ 8 and similarly for depression and a total score ≥ 11 may reflect an adjustment disorder in general, although due to anxiety or depression.	S18	No
Suicide ideation from Beck’s Depression Inventory (BDI-II)	Suicide risk.	None.	1	Arranged on a 4-point Likert scale as follows: 0 (“I have no thoughts of killing myself”), 1 (“I have thoughts of killing myself, but I wouldn’t do it”), 2 (“I would like to kill myself”), and 3 (“I would kill myself if I had the chance”).	S18	No
Abridged Job Descriptive Index (JDI)	Overall job satisfaction.	Job, compensation, promotion, supervision, and co-workers.	8	None.	S20	No
The Patient Health Questionnaire (PHQ-9)	Depression.	None.	8	It is organized on a 4-point Likert scale (0 = never to 3 = almost every day). Cutoff is a PHQ-9 score > or = 10.	S19–S22	No.
The Generalized Anxiety Disorder Scale (GAD-7)	Anxiety.	None.	7	It is organized on a 4-point Likert scale (0 = never to 3 = almost every day). Cutoff is a PHQ-9 score > or = 10.	S19–S21	No
The Brief Resilience Scale (BRS)	Resilience and ability to recover from adversity.	None.	6	Organized in a 5-point Likert scale to assess the extent to which the interviewed agreed to give a statement, in which 1 means completely disagree and 5 means completely agree.	S20, S21, and S23	No
The Perceived Stress Scale (PSS)	Stress.	None.	10	It is organized in a Cohen scale (0, never; 1, almost never; 2, sometimes; 3, quite often; 4, very often) to score the scale, A high score indicates a high perception of stress.	S4, S20–S22	No.
Self-Reporting Questionnaire (SRQ-20)	Minor psychiatric disorders.	Anxious and depressed mood, somatic mood, decreased energy, and depressive thoughts.	20	If the values are equal to or greater than 7 points, or a greater proportion of positive responses in both sexes, based on a study with police officers, then this indicates minor psychological problems.	S28	No.
Symptom Checklist 90 (SCL-90)	Psychological suffering and symptoms of psychopathology.	Somatization, obsessive-compulsive, interpersonal sensitivity, depression, anxiety, hostility, phobic anxiety, paranoid ideation, and psychosis.	90	If the number of positive numbers is superior to 43 points or the total score is superior to 160.	S29	No.
Copenhagen Psychosocial Questionnaire (COPSOQ)	Job satisfaction.	None.	7	Arranged on a four-point Likert scale, ranging from 1 = very satisfied to 4 = very unhappy. The cutoff points vary from 0 (lowest job satisfaction) to 100 (highest job satisfaction.	S26 and S27	No.
Police mental health ability (PMHA)	Occupational stress.	Cognitive intelligence, emotional catharsis, quick determination, behavioral impulse, and search for rewards.	20	It works with a “yes” or “no” response. The cutoff values are not mentioned.	S26	Yes.
The Impact of Event Scale—Revised (TIES-r)	Post-Traumatic Stress Disorder.	Intrusion, avoidance, and hypervigilance.	22	A 5-point Likert scale, ranging from 0 (“not at all”) to 4 (“extremely”). The sum of the three subscale scores determines a composite PTSD score for which the typical clinical threshold for the presence of PTSD is a score ≥ 33.	S18 and S24	No

Source: Elaborated by the authors, 2024.

**Table 4:** Psychometric properties of the identified instruments (*n* = 27).

Instrument	Cronbach’s Alpha	Test–Retest	Item-item Correlation
Brief COPE	0.85	0.71	
Brief Resilience Scale (BRS)	0.8	0.69	
Copenhagen Psychosocial Questionnaire (COPSOQ)	0.82		
DECORE-21	In subscales: 0.6 in cognitive demands, 0.78 in control, 0.84 in organizational support, and 0.92 in rewards.		
Effort-Reward Imbalance Questionnaire (ERIQ)	In subscales: effort with 0.78, reward with 0.91, and overcommitment with 0.81.		
Generalized Anxiety Disorder Scale (GAD-7)	0.89	0.83	0.65
Granada Burnout Questionnaire (GBQ)	In subscales: emotional exhaustion = 0.87, depersonalization = 0.85, and personal accomplishment = 0.8		
Hospital Anxiety and Depression Scale (HADS)	0.78 and 0.73 for the anxiety and depression subscales.		
Job Descriptive Index (JDI)	0.85		
Job Stress Survey (JSS)	For the severity and frequency of job pressure, the scores were 0.83 and 0.85, whereas for the severity and frequency of lack of support, the scores were 0.83 and 0.85, respectively.		
Law Enforcement Officer Stress Survey (LEOSS)	0.87	0.67	
Maslach Burnout Inventory (MBI)	0.87, 0.73, and 0.80 for the exhaustion, depersonalization, and achievement subscales		
Norwegian Police Stress Survey (NPSS)	For the severity and frequency of serious operational tasks, the scores were 0.82 and 0.83. For the severity and frequency of work injuries, the scores were 0.84 and 0.76, respectively.		
Operational Police Stress Questionnaire (PSQ-OP)	0.94		0.7
Organizational Police Stress Questionnaire (PSQ-Org)	0.94		0.72
Patient Health Questionnaire (PHQ-9)	0.9	0.94	
Perceived Stress Scale (PSS)	0.87	0.86	
Police mental health ability (PMHA)	0.863	0.73	
Police Role Expectations (PRE)	0.77		
Psychological Injury Risk Indicator (PIRI)	0.83	0.64	
Self-Reporting Questionnaire (SRQ20)	0.84		
Spanish Burnout Inventory (SBI)	In subscales: work excitement = 0.64; mental exhaustion = 0.85; guilt = 0.79; and indolence = 0.71		0.7
Suicide ideation from Beck’s Depression Inventory (BDI-II)	0.85		
Symptom Checklist 90 (SCL-90)	0.93		
The Impact of Event Scale—Revised (TIES-r)	0.92, 0.83, and 0.85 for the intrusion, avoidance, and hypervigilance subscales	0.86	
V. Bokyo Burnout Inventory (VBBI)	0.94		0.71
Work and Well-Being Assessment for Police (WWBAP)	0.74–0.86 in subscales		0.7

Source: elaborated by the authors, 2024.

## Data Availability

The data presented in this study are available on request from the corresponding author.
